# Altering carbon allocation in hybrid poplar (*Populus alba × grandidentata*) impacts cell wall growth and development

**DOI:** 10.1111/pbi.12682

**Published:** 2017-03-04

**Authors:** Faride Unda, Hoon Kim, Charles Hefer, John Ralph, Shawn D. Mansfield

**Affiliations:** ^1^ Department of Wood ScienceUniversity of British ColumbiaVancouverBCCanada; ^2^ Department of BiochemistryUniversity of WisconsinMadisonWIUSA; ^3^ Department of Energy Great Lakes Bioenergy Research CenterWisconsin Energy InstituteMadisonWIUSA; ^4^ Biotechnology PlatformAgricultural Research CouncilPretoriaSouth Africa

**Keywords:** galactinol synthase, RFOs, tension wood, cellulose, lignin, sugar signalling

## Abstract

Galactinol synthase is a pivotal enzyme involved in the synthesis of the raffinose family of oligosaccharides (RFOs) that function as transport carbohydrates in the phloem, as storage compounds in sink tissues and as soluble metabolites that combat both abiotic and biotic stress in several plant species. Hybrid poplar *(Populus alba × grandidentata*) overexpressing the *Arabidopsis thaliana GolS3* (*AtGolS3*) gene showed clear effects on development; the extreme overexpressing lines were stunted and had cell wall traits characteristic of tension wood, whereas lines with only moderate up‐regulation grew normally and had moderately altered secondary cell wall composition and ultrastructure. Stem cross‐sections of the developing xylem revealed a significant increase in the number of vessels, as well as the clear presence of a G‐layer in the fibres. Furthermore, *AtGolS3‐OE* lines possessed higher cellulose and lower lignin contents, an increase in cellulose crystallinity, and significantly altered hemicellulose‐derived carbohydrates, notably manifested by their mannose and xylose contents. In addition, the transgenic plants displayed elevated xylem starch content. Transcriptome interrogation of the transgenic plants showed a significant up‐regulation of genes involved in the synthesis of *myo*‐inositol, along with genes involved in sucrose degradation. The results suggest that the overexpression of GolS and its product galactinol may serve as a molecular signal that initiates metabolic changes, culminating in a change in cell wall development and potentially the formation of tension wood.

## Introduction

Optimizing carbon assimilation within an array of dynamic environmental stimuli plays a vital role in shaping the development, fitness and survival of plants. Across species, it has been shown that there is an evolutionarily conserved relationship between leaf photosynthetic capacity and plant biomass investment in leaf area (Reich *et al*., 1997). Sucrose is central to plant metabolism, and it is the dominant metabolite involved in the growth and development of plant cells. It is the primary product of photosynthesis; in vascular plants, the metabolite is most commonly translocated to sink organs where it provides the carbon for a variety of biosynthetic reactions. When imported into sink tissues, the influx of sucrose is crucial for the maintenance of cellular metabolism, respiration and cell wall biosynthesis, and can be converted to starch for storage and use at a later time (Canam *et al*., 2008a,b; Coleman *et al*., 2008).

Given that source tissue (i.e. mature leaves) governs the availability of carbohydrates essential for plant development and growth, it is fair to postulate that the products of primary metabolism are therefore intricately involved in mediating plant development. However, the mechanism by which plants sense changes in the availability of carbohydrates, and consequently how this in turn modulates growth, is not fully known for all development processes, especially cell wall development. Sucrose, and likely other carbohydrate moieties, can serve as a signal molecule that regulates gene expression (Leplé *et al*., 2007; Reich *et al*., 1997; Sturm, 1999) and consequently influences associated metabolic pathways, ultimately affecting morphological development (Coleman *et al*., 2006; Kutschera and Heiderich, 2002). Substantial information exists regarding the flux of sucrose in leaves in relation to photosynthesis, including kinetics and feedback inhibition data on many of the genes and proteins involved in its synthesis (Maloney *et al*., 2015; Park *et al*., 2008). Transcriptionally, the genes encoding these enzymes often coincide with developmental state (Barratt *et al*., 2009; Canam *et al*., 2006, 2008a,b), especially in developing leaves transitioning from sink to source, as well as during the mobilization of carbohydrates during phenological events, and in fruit development. Sucrose synthesis is also strongly influenced by post‐transcriptional events such as phosphorylation, which is known to influence the rate of synthesis, as well as protein–protein interactions (Kutschera and Heiderich, 2002). However, very little is known about the regulatory control of sucrose anabolism and the proteins that manifest such control. Given that glycolytic enzymes have been shown to function as transcription factors (Tsai *et al*., 1997), the possibility exists that the sucrolytic enzymes themselves may inherently exhibit dual functionality similar to hexokinase, which has been shown to function both as a key enzyme in primary metabolism and as a sensor of hexose sugars that inform plant resource allocation (Miller *et al*., 2000; Wiese *et al*., 2004). Recent evidence suggests that the majority of starch degradation at night occurs through the hydrolytic pathway, resulting in the formation of maltose and glucose in the cytosol (Raines and Paul, 2006). This opens up the possibility that altered starch:sucrose ratios can be sensed by tracking flux through the cytosol (Sharkey *et al*., 2004). This mechanism is likely to form part of a complex regulatory network, involving several carbohydrate molecules, that maintains a balance between carbon storage and utilization. How such changes in carbon balance are then linked to changes in development is not known, but likely play a central role in the process of integrating the effects of light, hormones and nutrient status (Rolland and Sheen, 2005) and, ultimately, plant growth and cell wall deposition.

*Myo*‐inositol is a multipurpose compound that generates several derivatives including phosphatidylinositols (PtdIns), inositol polyphosphates, D‐pinitol, cell wall polysaccharides and compatible solutes such as the raffinose family of oligosaccharides (Valluru and Van den Ende, 2011). For example, the PtdIns act both as structural membrane lipid molecules and as signals, where one such derivative, InsP3, has been implicated in the early signalling of gravity sensing and the initiation of a gravitropic response in oat pulvinus and Arabidopsis (Perera *et al*., 2001, 2005). Similarly, the lipid‐dependent InsP6 has been hypothesized to act as a signalling molecule to release Ca^2+^ and activate signalling pathways (Munnik and Vermeer, 2010). Alternatively, the lipid‐independent derivative InsP6 is the main phosphorous storage compound in sink tissues that can be later dephosphorylated to InsPs and Ins that are involved in the synthesis of compatible solutes (RFOs, galactinol) when the plant is under stress (Munnik and Vermeer, 2010).

Members of the RFOs serve as transport carbohydrates in the phloem, storage compounds in sink tissues, and metabolic agents to combat both abiotic and biotic stress in several plant species (Cunningham *et al*., 2003; Kim *et al*., 2008; Panikulangara *et al*., 2004; Philippe *et al*., 2010; Taji *et al*., 2002). Although there is indirect evidence supporting a signalling role for galactinol and RFOs under biotic and abiotic stress, the exact mechanism and its direct targets remain unclear. In this study, we test the hypothesis that cell wall attributes and plant development can be altered by augmenting the available soluble sucrose pools by examining the effects of altering carbon allocation in hybrid poplar by overexpressing exogenous galactinol synthase (GolS). Several lines were only marginally affected and, in many cases, had improved cell wall characteristics; however, two transgenic lines possessing the highest transcript abundance in the phloem clearly displayed significant developmental effects. Additionally, these transgenic lines possessed wood chemistry and fibre traits characteristic of tension wood, such as lower total lignin content, higher cellulose content, xylem that often contains small vessels and fibres with a unique inner cell wall layer (G‐layer) primarily made of cellulose (Telewski 2006). Together, this implies that GolS, or its product (galactinol) may have a dual role, acting both as a metabolite or as a signal that impacts cell wall development and the transition from normal wood to a specialized secondary cell wall.

## Results

### Generation of transgenic poplar trees

The full‐length coding sequence of the *A. thaliana GolS3* (At1g09350) gene (Taji *et al*., 2002) was cloned and inserted into the binary vector pSM‐3 driven by a universal 35S promoter. *Agrobacterium*‐mediated transformation of hybrid poplar yielded eleven *AtGol3‐OE* transformants that were verified by genomic screening. After 7 weeks of growth in tissue culture, RNA was isolated and used to generate cDNA using reverse transcriptase. Transcript abundance was then determined by real‐time PCR (RT‐PCR). Subsequently, ten lines possessing the highest transgene transcript abundances, based on RT‐PCR, were clonally propagated and eventually transferred to the UBC greenhouse.

**Figure 1:**
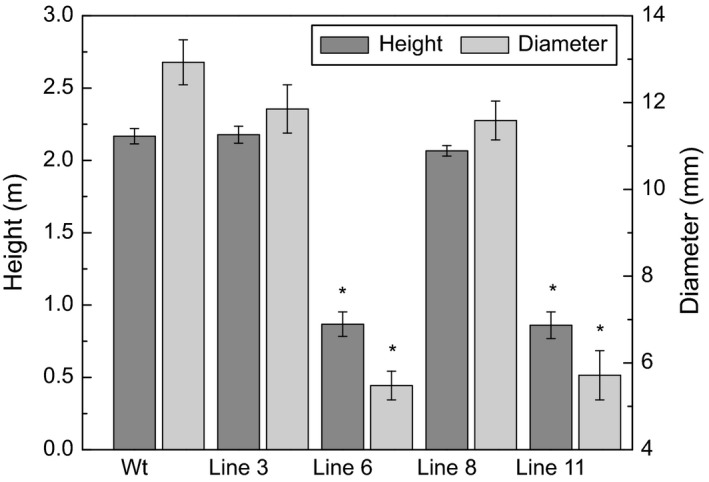
Height and diameter. Height from the base of the stem to the apex, and diameter 10 cm from the base of the stem of 5‐month‐old greenhouse‐grown *AtGolS3‐OE* and wild‐type hybrid poplar. Bars represent the standard error of the mean (n = 10). Asterisks represent statistical difference from wild type (wt) at 95% level.

**Figure 2:**
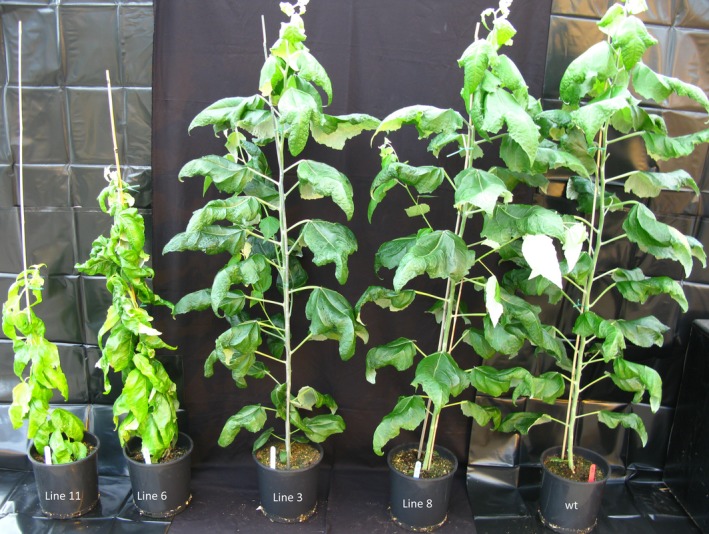
Transgenic hybrid poplar phenotype. Five‐month‐old greenhouse‐grown transgenic *AtGolS3* hybrid poplar and wild‐type trees.

After 5 months of growth, the height and diameter of the stems were recorded (Figure 1). Among the 10 transgenic lines, lines 6 and 11 showed distinct growth phenotypes (Figure 2); specifically, they were severely stunted in height (0.86 m and 0.86 m, respectively) and diameter (5.47 mm and 5.71 mm, respectively) compared with the wild‐type trees (2.16 m in height and 12.92 mm of stem diameter), and displayed a distinct vine‐line appearance. The remaining lines appeared similar in stature and architecture to wild‐type trees; therefore, two transgenic lines (3 and 8) were selected for in‐depth characterization along with the two lines that were visually distinct from the untransformed control trees.

**Figure 3:**
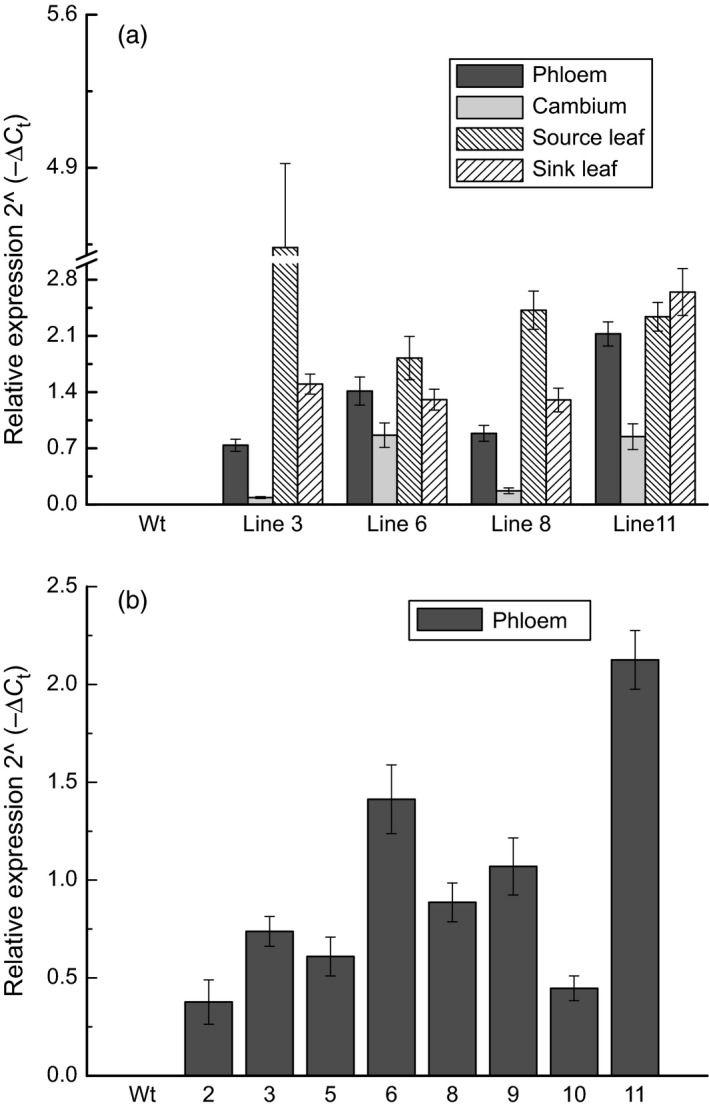
Transcript abundance. *Arabidopsis thaliana* galactinol synthase 3 gene (*AtGolS3*) (a) in 4 tissues of selected lines and (b) in phloem tissue of eight lines of 5‐month‐old greenhouse‐grown hybrid poplar. Expression is relative to the translation initiation factor 5A (*TIF5A* = reference gene) using the formula 2^−∆Ct^. Bars represent the standard error of the mean (n = 3).

Relative transcript abundance was again examined on the greenhouse‐grown trees; transcripts were clearly detected in all four *AtGolS3‐OE* lines selected for in‐depth examination, but not in the wild type, as expected (Figure 3a). Lines 6 and 11 had greater transgene abundance in the phloem and developing xylem than the other two transgenic lines (3 and 8). The expression was different in source leaves where the non‐phenotypic lines (lines 3 and 8) displayed more transcript than lines 6 and 11. Transgene expression was also examined in the phloem of other transgenic lines (1, 2, 4, 5, 7, prior to selection for in‐depth growth and cell wall phenotyping; Figure 3b). Among all lines, lines 6 and 11 consistently showed the highest transgene expression.

### Soluble sugar and starch analyses

**Figure 4:**
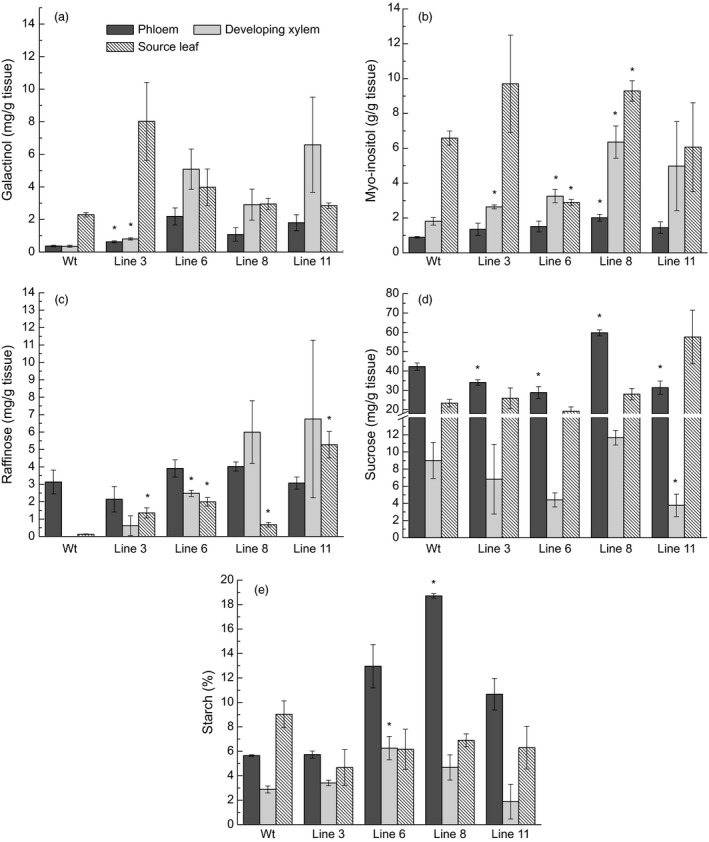
Non‐structural carbohydrates. Concentration (mg/g of dry tissue) of (a) galactinol, (b) *myo*‐inositol, (c) raffinose, (d) sucrose, and (e) starch (% of dry weight) in tissues of 5‐month‐old greenhouse‐grown wild‐type and *AtGolS3‐OE* hybrid poplar. Bars represent the standard error of the mean (n = 3). Asterisks represent lines statistically different from wild type (wt) at the 95% level.

Soluble sugars (galactinol, *myo*‐inositol, sucrose and raffinose) were analyzed in phloem, developing xylem and source leaves of wild type and the four *AtGolS3‐OE* lines. Galactinol concentration increased in all *AtGolS3‐OE* lines compared with wild‐type trees in all tissues, with lines 6 and 11 displaying the highest concentration in the phloem and developing xylem tissues (Figure 4a). *Myo*‐inositol, which is one of the substrates involved in the reaction catalyzed by galactinol synthase, was higher in all transgenic lines in the phloem and developing xylem, and lower in lines 6 and 11 in leaf tissue compared with the wild‐type trees (Figure 4b). Only trace amounts of raffinose were present in the developing xylem of wild‐type trees, while it was apparent in all transgenic lines, in all tissues (Figure 4c). Sucrose content was lower in lines 6 and 11 in the phloem and developing xylem compared with the other transgenic lines and wild‐type trees (Figure 4d).

**Figure 5:**
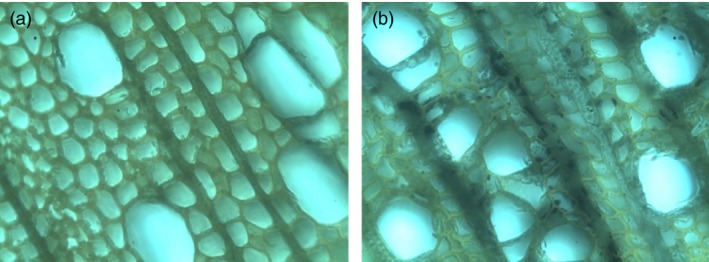
Starch in xylem tissue. Staining with iodine‐blue of (a) wild type and (b) *AtGolS3‐OE* line 6. Transgenic lines showed starch granules on the xylem rays.

Interestingly, the overall amount of sucrose in the phloem of the wild‐type hybrid poplar trees was 13 times higher than the amount of raffinose, confirming that sucrose is the major transporting sugar in poplar (Russin and Evert, 1985; Slewinski *et al*., 2013). Starch was also reduced in source leaf tissue in all transgenic lines (Figure 4e), whereas phloem starch was higher in all transgenic lines (significantly higher in line 8) compared with wild type, suggesting that carbon partitioning was substantially augmented by *GolS* overexpression in these transgenic trees. Cross‐sections of transgenic and wild‐type trees, stained with Lugol's solution, clearly showed that starch was accumulating as discernible starch granules in the rays of *AtGolS3‐OE* trees (Figure 5). The biochemical and histochemical findings were supported by whole‐cell NMR analysis which showed that the developing xylem was enriched in starch in the transgenic lines.

### Carbohydrate and lignin analysis

**Figure 6:**
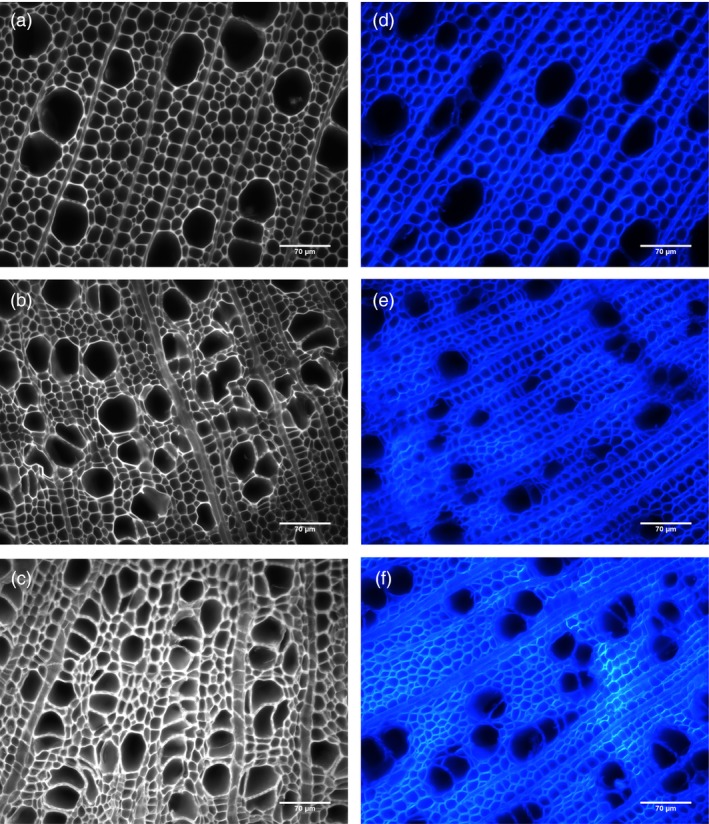
Autofluorescence and calcofluor staining. Autofluorescence (a–c) and calcofluor (d–f) staining of (a, d) wild type, (b, e) *AtGolS3‐OE* line 6, and (c, f) line 11 hybrid poplar. Transgenic lines show an irregular vessel phenotype and increased cellulose staining with calcofluor‐white (Scale bars: 70 μm).

Structural carbohydrate analysis showed that *AtGolS3*‐*OE* lines 6 and 11 were significantly different from wild‐type trees (Table 1), exhibiting higher arabinose and galactose levels, whereas rhamnose, xylose and, more significantly, mannose concentrations were reduced. The most remarkable change was an increase in glucose content in all transgenic lines, suggesting higher cellulose content. In accordance with the chemical analysis, cellulose‐specific histochemical staining with calcofluor‐white of stem cross‐sections (Figure 6) showed increased fluorescence. The total lignin content of the transgenic lines was concurrently decreased (Table 1).

**Table 1:** Structural cell wall carbohydrates and total lignin content of 5‐month‐old wild‐type and *AtGolS3‐OE* hybrid poplar trees. Values represent the mean and standard error of the mean in parentheses with bold values corresponding to a statistical difference from wild type (wt) at the 95% level (n = 3)

Lines	Arabinose, μg/mg	Rhamnose, μg/mg	Galactose, μg/mg	Glucose, μg/mg	Xylose, μg/mg	Mannose, μg/mg	Lignin, %
Wt	3.12 (0.48)	6.63 (0.52)	8.65 (0.67)	466.16 (6.82)	161.37 (4.58)	24.64 (0.94)	24.79 (0.21)
line 3	3.00 (0.40)	6.37 (1.79)	10.80 (1.16)	**509.43 (10.59)**	151.57 (5.62)	**29.02 (0.84)**	23.06 (1.27)
line 6	**7.52 (0.71)**	4.63 (1.03)	**18.61 (2.79)**	**540.62 (23.77)**	**127.97 (4.87)**	**8.73 (0.80)**	**18.23 (0.40)**
line 8	3.12 (0.66)	5.28 (0.12)	10.06 (1.04)	481.09 (9.80)	151.54 (5.66)	22.54 (0.81)	23.58 (0.38)
line 11	**8.67 (1.30)**	7.15 (1.35)	**17.02 (0.97)**	**509.45 (4.27)**	**134.61 (5.25)**	**11.39 (1.44)**	**19.38 (0.47)**

**Figure 7:**
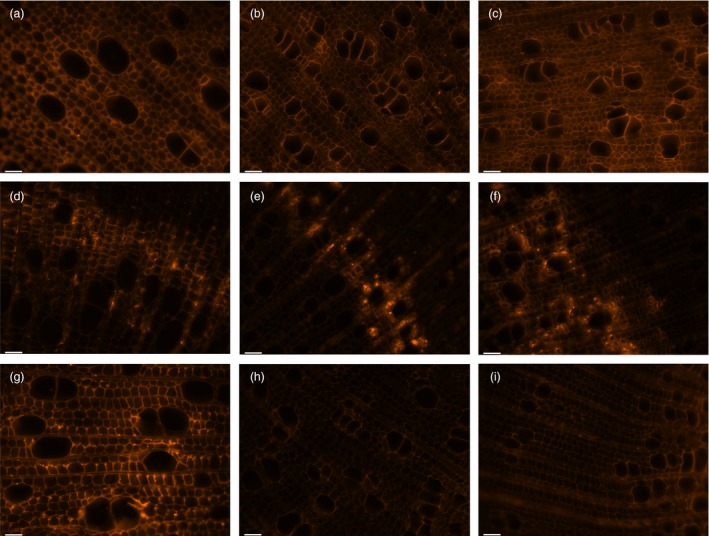
Immunofluorescence labelling of xylem tissue. (a, d and g) Wild type, (b, e and h) *AtGolS3‐OE* line 6, and (c, f and i) * AtGolS3‐OE* line 11 hybrid poplar. Tissue was labelled with the anti‐xylan LM10 antibody (a–c); the anti‐RGI CCRC‐M7 antibody (d–f) and the anti‐mannan antibody (G‐I). Bars: 40 μm.

Microscopic observations of stem cross‐sections of the *AtGolS3‐OE* lines 6 and 11 showed irregular and smaller vessels, increased number of vessels per unit area (Table 2) and increases in the amount of tissue that appeared to be tension wood (Figure 6, fibres visually more stained with calcofluor, with a gelatinous layer; G‐layer) when compared with wild‐type trees. To further analyze the change in structural carbohydrate content, immuno‐labelling of cross‐sections with antibodies LM10 (anti‐xylan), CCRC‐M7 (anti‐rhamnogalacturonan I) and anti‐(1‐4)‐β‐mannan was used to evaluate lines 6 and 11 relative to wild type. The anti‐xylan antibody LM10 bound evenly to xylem tissue (fibres, vessels and ray parenchyma; Figure 7a–c). Fluorescence was similar in both the transgenic lines and wild type, but binding of the antibody was higher in areas of ‘tension wood’ in all samples and with all antibodies, especially with CCRC‐M7 (RGI) (Figure 7d–f). The anti‐β‐(1‐4)‐D‐mannan binding was noticeably stronger in wild‐type tissue compared with lines 6 and 11 (Figure 7g–i), as is consistent with the substantial reduction in mannose shown by cell wall analyses. In addition, it appears that in wild‐type sections the anti‐mannan binding was enhanced in fibre cell walls when compared to vessels.

**Table 2:** Vessel count and quantification of cross‐sections of 5‐month‐old greenhouse‐grown wild‐type and *AtGolS3‐OE* trees. Values represent the mean and standard error of the mean in parentheses with bold values corresponding to a statistical difference from wild type (wt) at the 95% level (n = 3)

Lines	Area per vessel (μm^2^)	Number (per 100 000 μm^2^)	Length (μm)	Width (μm)
wt	1536.02 (50.03)	9.99 (0.40)	48.73 (1.45)	35.22 (1.84)
line 6	**544.47 (16.31)**	**21.44 (1.87)**	**30.07 (0.82)**	**24.91 (0.78)**
line 8	1427.88 (54.416)	**8.17 (0.48)**	**44.61 (1.27)**	33.96 (0.92)
line 11	**338.83 (10.49)**	**23.00 (2.14)**	**20.15 (0.78)**	**17.76 (0.68)**

**Figure 8:**
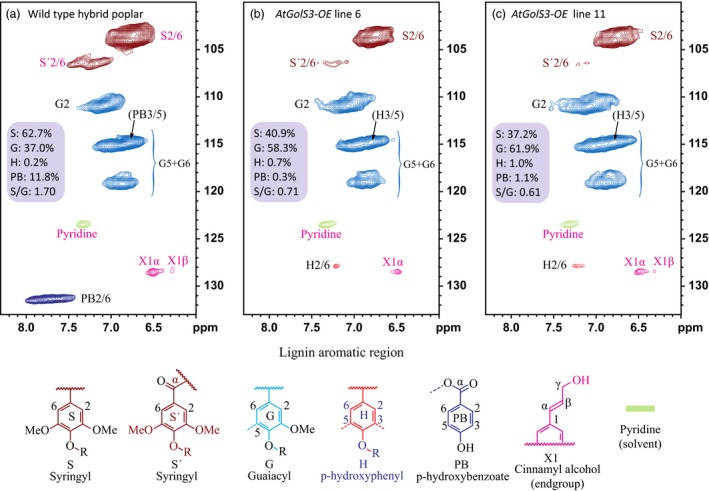
Lignin aromatic regions. 2D ^1^H–^13^C correlation (HSQC) NMR spectra from whole‐cell wall gels in DMSO‐d_6_/pyridine‐d_5_ (4:1, v/v). (a) Wild‐type *Pa×g* hybrid poplar, (b) *AtGolS3‐OE* line 6, (c) *AtGolS3‐OE* line 11. Correlations from the aromatic C/H pairs are well dispersed and can be categorized by the type of aromatic units (syringyl S, guaiacyl G, *p*‐hydroxyphenyl H, *p*‐hydroxybenzoate PB); colour coding is according to the structures shown.

*AtGolS3‐OE* lines 6 and 11 and wild‐type trees were also analyzed by 2D NMR to study the distribution of the anomerics and lignin without substantially degrading the cell wall polymeric structures. The lignin monomer distribution and abundance did not appear to be altered significantly between the transgenic lines and wild‐type trees; however, the syringyl content was slightly reduced. A striking difference in the lignin was observed, as the *p*‐hydroxybenzoate moieties, which commonly acylate the lignin polymer side‐chain γ‐OHs, were completely absent in the transgenic lines (Figure 8).

**Figure 9:**
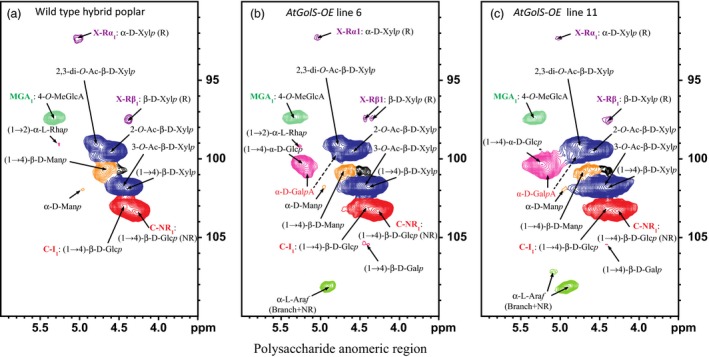
Polysaccharide anomeric regions. 2D ^1^H–^13^C correlation (HSQC) NMR spectra from whole‐cell wall gels in DMSO‐d_6_/pyridine‐d_5_ (4:1, v/v). (a) Wild‐type *Pa×g* hybrid poplar, (b) *AtGolS3‐OE* line 6, (c) *AtGolS3‐OE* line 11. β‐D‐Glc*p*, β‐D‐glucopyranoside; α‐D‐Man*p*, α‐D‐mannopyranoside; β‐D‐Man*p*, β‐D‐mannopyranoside; β‐D‐Xyl*p*, β‐D‐xylopyranoside; α‐L‐Ara*f*, α‐L‐arabinofuranoside; α‐L‐Rha*p*, α‐L‐rhamnopyranoside; 2‐*O*‐Ac‐β‐D‐Xyl*p*, acetylated β‐D‐Xyl*p*; 3‐*O*‐Ac‐β‐D‐Xyl*p*, acetylated β‐D‐Xyl*p*; 4‐*O*‐MeGlcA, 4‐*O*‐methyl‐α‐D‐glucuronic acid; α‐D‐Gal*p*A, α‐D‐galacturonic acid. R, reducing end; NR, non‐reducing end.

Arabinose (α‐L‐Ara*f*) was readily evident in small amounts in the NMR spectra of the transgenic lines, but not in wild‐type trees. Consistent with wet chemical analysis, mannose (β‐D‐Man*p*) was considerably reduced (Figure 9), and α‐linked glucose [(1‐4)‐α‐D‐Glc*p*], that is indicative of starch, appeared in the transgenic lines but was largely absent in the wild‐type spectra.

### Xylem characterization

**Figure 10:**
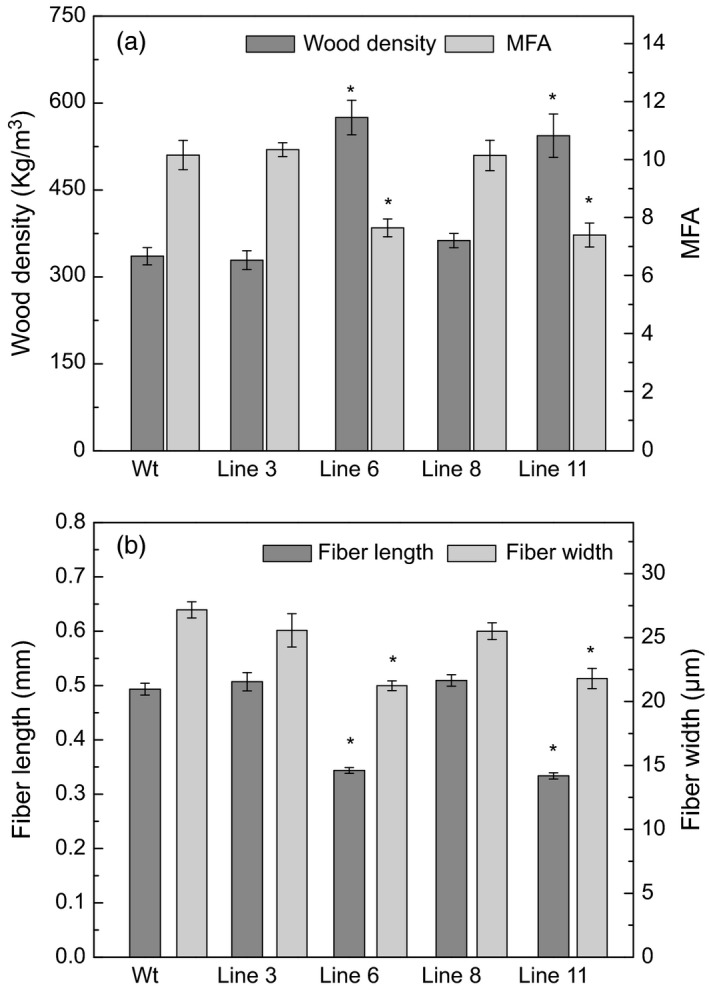
Wood physical properties. (a) Wood density and microfibril angle (MFA) and (b) fibre length and width of 5‐month‐old greenhouse‐grown wild‐type and *AtGolS3‐OE* hybrid poplar. Bars represent the standard error of the mean (n = 9). Asterisks represent samples statistically different from wild type (wt) at the 95% level.

The xylem properties of the *AtGolS3‐OE* lines and wild‐type trees were compared. Most of the transgenic lines had higher wood densities, with lines 6 and 11 being substantially higher (575.04 and 543.6 kg/m^3^, respectively) than the wild type (335.8 kg/m^3^). Lines 3 and 8 also had increased wood densities, showing average densities of 328.9 and 362.78 kg/m^3^ (Figure 10a). The wood microfibril angle (MFA) was reduced in lines 6 and 11 (7.69° and 7.44°) when compared with either wild‐type trees (10.20°) or the other transgenic lines examined (10.39° and 10.19° in lines 3 and 8, respectively) (Figure 10a). The per cent cell wall crystallinity also increased in the same lines (51.3% and 55%, respectively) compared with either wild‐type poplar (46.7%) or transgenic lines 3 and 8 (46.0% and 44.7%; Table 3). In addition, based on the fibre length measurement, fibres of lines 3 and 8 were longer than fibres of wild‐type plants as well as lines 6 and 11 (Figure 10b).

**Table 3:** Crystallinity of 5‐month‐old greenhouse‐grown wild‐type and *AtGolS3‐OE* hybrid poplar trees. Values represent the mean and standard error of the mean in parentheses with bold values corresponding to a statistical difference from wild type (wt) at the 95% level (n = 3)

Lines	Crystallinity, %
wt	46.7 (1.2)
line 3	46.0 (1.5)
line 6	51.3 (3.4)
line 8	44.7 (0.9)
line 11	**55.0 (3.4)**

### mRNA‐Seq

**Figure 11:**
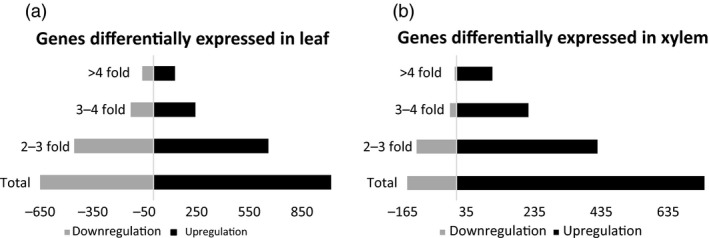
RNA‐Seq. Transcriptional response of the overexpression of the *AtGolS3* in hybrid poplar. (a) Differentially expressed genes in leaf tissue and (b) differentially expressed genes in developing xylem.

mRNA‐Seq data were generated and analyzed from source leaf and developing xylem tissue of 5‐month‐old greenhouse‐grown wild type and *AtGolS3‐OE* line 6. In the leaf, 1655 transcripts were differentially expressed that had at least a twofold change when compared with wild‐type trees of the same age and grown under similar conditions; 61% of the genes were up‐regulated. Additionally, in the developing xylem, 890 transcripts were differentially expressed with a minimum twofold change, and 83% of transcripts were up‐regulated. The overexpression of the *AtGolS3* gene had the largest effect at the transcriptional level in leaf tissue compared to the developing xylem (Figure 11).

Differentially expressed genes were uploaded to MapMan to visualize the altered expression in the various biological pathways (Figure S1). In leaf tissue, several transcripts involved in photosynthesis and photorespiration showed down‐regulation in the transgenic lines, whereas genes related to sucrose degradation and inositol metabolism were up‐regulated in leaf tissue. Clearly, altering available sucrose in the sink tissue has a feedback response that triggers a reduction in sucrose production and an increase in sucrose re‐synthesis. Table S1 shows an overview of metabolism by pathways: major CHO metabolism (synthesis and degradation of sucrose and starch), minor CHO metabolism (RFOs, *myo*‐inositol and callose), signalling (in sugar and nutrient physiology, phosphoinositides and receptor kinases) and hormone metabolism (auxin).

## Discussion

The raffinose family of oligosaccharides has been implicated in several intricate plant biological processes, including desiccation tolerance in seeds (Downie *et al*., 2003; Liu *et al*., 1995), transport of carbohydrates in the phloem (Volk *et al*., 1996) and protection of plants against a variety of biotic stress scenarios (Philippe *et al*., 2010; Taji *et al*., 2002). In poplar trees specifically, it has been suggested that the galactinol synthase genes play a role in the storage and seasonal mobilization of carbohydrates, in addition to a role in protecting the trees against biotic and abiotic stress (Unda *et al*., 2012). To further understand the specific function of this gene *in planta*, we studied the changes in the development of sink tissues caused by the overexpression of galactinol synthase in hybrid poplar. Several cell wall characteristics were altered in the transgenic plants, including cell wall density, MFA, tension wood formation, hemicellulose content, cellulose content and crystallinity, and lignin content and composition.

Plant development relies on intricate signalling systems that deliver information on the existing internal and external conditions. This information includes the status of cellular metabolites such as sugars, which are essential to development, and also participate in complicated signalling systems that respond to metabolite concentrations (Hanson and Smeekens, 2009). These regulatory networks and signalling systems are vital not only to development and growth, but also for the interaction with pathogens and abiotic stress conditions. The altered wood phenotype of the transgenic trees suggests that the overexpression of galactinol synthase or its resulting product (galactinol) may play an important role as a signal molecule to initiate a complex array of metabolic changes aimed at combating global stress. Consequently, it may also be involved in the initiation of tension wood formation, a specialized cell wall alteration known to occur in response to environmental stimuli, such as the gravitropic response that results in its reorientation to the upright position (Gerttula *et al*., 2015).

*Myo*‐inositol (Ins) and its derivatives (e.g. Ins‐derived galactinol and RFOs) play important roles in plant stress tolerance, and function either as metabolomic mediators or signalling molecules in various stress response pathways (Valluru and Van den Ende, 2011). For example, Perera *et al*. (2001) showed an increased concentration of Ins3P (generated by MIPs) in the lower half of the pulvini at the establishment of tissue polarity (during the gravitropic response) in oat shoots, and suggested that this provided the positional information in the pulvini prior to the redistribution of the auxin. Furthermore, it was suggested that actin‐dependent relocalization of PIN3, in response to gravity, provides a mechanism for redirecting auxin flux to trigger asymmetric growth (Friml *et al*., 2002). In poplar, PIN3 localizes to the endodermal cells containing starch‐filled amyloplast that sediments in response to gravity, suggesting that these are the gravity‐perceiving cells in the stem (Gerttula *et al*., 2015). Here, we show that the *AtGolS3* transgenic lines also accumulate starch bodies in the rays. Moreover, these same trees also show up‐regulation of PIN3 transcripts (mRNA‐Seq, Table S1), suggesting that the transgenic plants sense changes in the soluble Ins flux (several transcripts of the pathway were up‐regulated MIPs, MIOXs, Table S1), initiating a cascade of signalling responses resulting in the altered xylem phenotype. These findings concur with previous studies that implicated galactinol as a signal molecule in plants subjected to biotic stress (Cho *et al*., 2010; Kim *et al*., 2008). Additionally, Valluru and Van den Ende (2011) presented a model suggesting that galactinol and the RFOs maintain reactive oxygen species (ROS) homeostasis in plants, and proposed that these sugars may function as endogenous signals acting downstream of ROS signalling.

### Wood properties of highly up‐regulated transgenic lines are characteristic of tension wood

Cross‐sections of the extreme *AtGolS3* phenotypic lines showed smaller and irregular vessels compared with wild‐type trees. The number of vessels was significantly higher and a clear G‐layer was apparent in the fibres; the G‐layer serves as a characteristic of tension wood formation in angiosperm trees (Mizrachi *et al*., 2015). Consistent with the visual observations, the MFA was significantly reduced and the cell wall crystallinity increased. In poplar, as in most temperate tree species, tension wood fibres are characterized by the existence of a G‐layer that has microfibrils that are arranged almost parallel to the cell's long axis (Faruya *et al*., 1970). In the current study, the MFA of two of the *AtGolS3‐OE* lines (6 and 11) was reduced by approximately 25% compared with wild‐type trees.

In addition to the ultrastructural characteristics, the cell wall mannose content was extremely reduced in the *AtGolS*3*‐OE* lines compared with wild‐type trees. Similar results were obtained in the tension wood of G‐fibres in aspen which showed a two‐ to threefold reduction in both mannose and 1,4‐β‐mannan (Hedenstrom *et al*., 2009). These biochemical findings are consistent with molecular findings, where the inherent levels of mannan biosynthesis‐related transcripts were reduced in differentiating fibres of poplar developing a G‐layer during tension wood formation (Andersson‐Gunneras *et al*., 2006). We also observed a significant increase in galactan content. Tension wood of other species, such as *Eucalyptus goniocalyx* (Schwerin, 1958), *Betula pubescens* and *B*. *verrucosa* (Gustafsson *et al*., 1952), and *Fagus sylvatica* (Meier, 1962), have been shown to exhibit up to two‐ to fourfold more galactose than normal wood. Kuo and Timell (1969) showed that the G‐fibre galactan in American beech largely consisted of β‐(1→4)‐ and β‐(1→6)‐linked galactopyranoside residues, with a smaller component of galacturonic acid and rhamnose, as the disaccharide 2‐*O*‐α‐D‐GalA*p*‐L‐Rha, suggesting the presence of rhamnogalacturonan I (RGI) (Azuma *et al*., 1983; Kuo and Timell, 1969; Meier, 1962). Another important finding from the current 2D NMR analysis was the strong possibility of the presence of galacturonic acid, concurring with the previous findings. Pectin polysaccharides containing (1→4)‐α‐D‐galacturonic acid are one of the major components of the middle lamella and primary cell wall but are also present in small proportions in secondary cell wall, especially those enriched in gelatinous type cells (Gorshkova *et al*., 2010). Additionally, galacturonic acid together with rhamnose and galactose was shown to be present in the alkali‐insoluble residue of flax fibre (Mooney *et al*., 2001) suggesting that the pectic galactan can be tightly bound to cellulose in the secondary cell wall.

Immunofluorescence analysis of the *AtGolS3‐OE* lines showed that the CCRC‐M7 antibody, which recognizes RGI, bound more intensely to areas where tension wood was found. Similarly, immunochemical studies in sweetgum (*Liquidambar styraciflua;* Hamamelidaceae) and hackberry (*Celtis occidentalis*; Ulmaceae) showed that a number of antibodies that recognize arabinogalactan proteins and RGI‐type pectin components bind to the G‐layer (Bowling and Vaughn, 2008). The fact that the tissue is labelled with CCRC‐M7 antibody, which recognizes RGI as well as both soluble and membrane‐bound AGPs, supports the notion that tension wood is not entirely composed of crystalline cellulose. The increase in cellulose content and the alteration of the hemicellulose composition, together with the reduction of lignin content, make this transgenic approach an ideal strategy for poplar and other biomass improvement for industrial processing, especially bioenergy applications.

### *p*‐Hydroxybenzoate groups associated with S lignin are absent from transgenic lines

The total lignin content of the *AtGolS3‐OE* plants was shown to be slightly, but significantly, reduced. More detailed analysis using 2D NMR showed that the most noticeable change in the lignin structure was the absence of the *p*‐hydroxybenzoate groups that typically acylate poplar lignins (Figure 8). The γ‐acylated G (guaiacyl) and S (syringyl) units have been identified in the lignins from several species. Although the lignins of grasses have γ‐*p*‐coumarate substituents on many lignin subunits, poplar, willow and palm have analogous γ‐*p*‐hydroxybenzoates acylating the primary γ‐OH (Landucci *et al*., 1992; Meyermans *et al*., 2000; Ralph, 2010; Smith, 1955). In our study, the 2D NMR showed a slight reduction in the S lignin in the galactinol synthase overexpressed lines and an extreme reduction of its associated *p*‐hydroxybenzoate, as it was no longer detectable by NMR spectroscopy. Lack of the pendant *p*‐hydroxybenzoate groups acylating the lignin sub‐units may result in lignin macromolecules that are less ‘bulky’, and therefore more amenable to packing within the highly aligned cellulose microfibrils that dominate the specialized xylem cells comprising the tension wood.

## Conclusion

Soluble sugars, similarly to hormones, can act as signals for the expression of genes involved in plant growth and metabolism (Rolland *et al*., 2006). For example, excess photosynthate in source tissues down‐regulates photosynthesis to maintain homeostasis. Additionally, changes in the carbon balance activate several responses, many of which are still unknown but are expected to be part of the plant's general growth and development.

Galactinol has been studied for its role as the galactosyl donor for the synthesis of raffinose and higher oligosaccharides (i.e. stachyose). The most noticeable phenotype in transgenic plants up‐regulated in galactinol synthase was in the xylem composition resembling tension wood formation. The plants appear to sense the changes in the leaf‐soluble carbon flux due to the augmented production of galactinol, and consequently alter the carbohydrate balance which is also reflected in the increase in starch in xylem tissue (rays). Comparison of the transcript abundance of *AtGolS3* transgenic lines and wild‐type plants demonstrated a clear up‐regulation of genes involved in sucrose degradation, as well as an up‐regulation of genes involved in the *myo*‐inositol pathway. Collectively, the results suggest that the overexpression of *GolS*, and concomitant elevation of its product galactinol, may serve as a molecular signal that initiates a cascade of metabolic changes for combating stress, culminating in the formation of tension wood in poplar trees. Because of the lower relative levels of lignin, and the higher levels of cellulose and C6‐sugars largely derived from hemicelluloses, in such transgenics, they appear to be better suited as plant substrates for biomass conversion than their wild‐type counterparts. Thus, controlling the level of expression in such transgenics, or using a xylem‐specific promoter, appears to be a suitable strategy to promote tension wood formation, with its commensurate increased cellulose deposition, without affecting agronomic traits for dedicated bioenergy crops such as poplar.

## Materials and methods

### Plasmid construction

The *A. thaliana GolS*3 (At1g09350), which was previously shown to be cold‐inducible (Taji *et al*., 2002), was cloned from Columbia ecotype (CO) cDNA using primers: *AtGolS3.Fw* 5′‐CGCGGATCCATGGCACCTGAGATGAACAACAAGTTG‐3′ and *AtGolS3.Rv* 5′‐CGCGAGCTCCTGGTGTTGACAAGAACCTCGCTC‐3′. The cloned *GolS* gene was ligated into the pSM3 vector (pCambia 1390 containing a double 35S promoter, Mansfield Lab, UBC) using the *BamHI* and *SacI* restriction enzymes. The vector was then subjected to genomic sequencing to confirm gene, frame and orientation, and then transformed into *Agrobacterium tumefaciens* C58 strain, which was used for plant transformation.

### Hybrid poplar transformations

*Populus alba × grandidentata* (P39) leaf discs were harvested from 4‐week‐old tissue‐culture‐grown plants using a cork borer. In all, 20 plates containing 25 leaf discs (7 mm^2^) per genotype were co‐cultivated with 30 mL of bacterial culture for 30 min at 28 °C in a gyratory shaker (100 rpm). Following co‐cultivation, the explants were blotted dry and placed abaxially on woody plant media (WPM) supplemented with 0.1 NAA, 0.1 μm BA and 0.1 μm TDZ culture medium. The plates were cultured in the dark for two days at room temperature. On the third day, residual *Agrobacterium* was eliminated by transferring the leaf discs to WPM containing 250 mg/L cefotaxime and 500 mg/L carbenicillin. Plates were kept in the dark for an additional 2 days. Explants were then transferred to WPM selection media containing 250 mg/L cefotaxime, 500 mg/L carbenicillin and 25 mg/L hygromycin. After emergence, only one shoot per leaf disc was excised and placed on WPM selection media. After 6 weeks growth, explants were transferred to fresh medium of the same composition, and permitted to develop. Plants were confirmed as transgenic by genomic DNA screening, and those identified as positive were then sub‐cultured and multiplied on antibiotic‐free WPM.

### Plant growth

Transgenic trees were multiplied in WPM until approximately eight plantlets of each transgenic event were of similar size, along with the appropriate control, non‐transformed trees. The trees were then moved to 2 gallon pots containing perennial soil (50% peat, 25% fine bark and 25% pumice; pH 6.0), and maintained on flood tables with supplemental lighting (16 h days) and watered daily with fertilized water in the greenhouse at UBC Vancouver, BC.

### Tissue collection

Source leaves were collected using the leaf plastochron index (PI = 5), where PI = 0 was defined as the first leaf greater than 5 cm in length and where PI = 1 is the leaf immediately below PI = 0. Sink leaves were defined as small, not fully expanded leaves. Phloem tissue included the bark, phloem tissue and cambial cells. Developing xylem corresponded to a slight scrapping of the stem immediately adjacent to the cambial zone.

### Genomic DNA extraction

The CTAB (Sigma‐Aldrich Co., San Louis, MO) extraction method was used to isolate hybrid poplar DNA. Briefly, the tissue was frozen with liquid N_2_ and ground to a powder with 1 mL extraction buffer (2% w/v CTAB, 1.4 m NaCl, 20 mm EDTA, 100 mm Tris–HCl, 1% PVP and 0.2% v/v β‐mercaptoethanol). The DNA concentration was measured using a spectrophotometer ND1000 (NanoDrop Technologies, Inc. DE). DNA was stored at −20 °C until further use.

### RNA isolation

Hybrid poplar RNA was isolated as per Kolosova *et al*. (2004), using source and sink leaves, phloem, and developing xylem tissue. The RNA concentration was calculated using a GeneQuant *pro* or a ND1000 spectrophotometer. DNase I DIGEST kit (Ambion Inc. Burlington, ON) was used to eliminate contaminating DNA, RNA was then quantified and 1 μg was used to generate cDNA using the SuperScript II RT kit (Invitrogen Canada Inc.). cDNA was stored at −20 °C.

### PCR analysis of putative transformants

Plants that survived antibiotic selection were evaluated for the presence of the transgene. Transgene detection was established by PCR screening of genomic DNA using gene‐specific primers: *AtGolS3scFw* 5′‐AGCCTCCCCACTTATTACAAC‐3′ and *AtGolS3scRv* 5′TGCACAGTAATGAACAACCTT‐3′.

### Real‐time PCR (RT‐PCR)

RT‐PCR reactions consisted of 12.5 μL of SYBR^®^ Green QRT‐PCR master mix (Invitrogen, Carlsbad, CA), 20 pmol of *AtGolS3scFw* and *AtGolS3scRv* primers (described above), 1 μL of cDNA and distilled deionized water to a total volume of 25 μL. RT‐PCR was performed on an Mx3000P^®^ QPCR System (Stratagene, La Jolla, CA) using the following thermal cycler conditions to amplify a 201 bp fragment of the *AtGolS3* transcript: 1 cycle of 5 min at 95 °C, 40 cycles of 95 °C for 30 s, 55 °C for 1 min and 72 °C for 30 s, followed by 1 cycle of 95 °C for 1 min, 55 °C for 30 s and 95 °C for 30 s. Relative expression was calculated using the following equation ∆ct = 2^−(ct target gene‐ct TIF5A)^, where *TIF5A* was used as the reference gene (Coleman *et al*., 2009).

### Growth measurements

After 4 months of greenhouse growth, tree height from root collar to the apex of the tree was recorded, and stem diameter was measured using digital calipers 10 cm above the root collar (soil level). *AtGolS3‐OE* lines and corresponding wild‐type trees were harvested after 5 months of growth, and tissues were kept at −80 °C until further use.

### Structural chemistry analysis

Dried wood samples from 5‐month‐old greenhouse‐grown trees were used to determine lignin and carbohydrate content following a modified Klason method described by Cullis *et al*. (2004).

### Soluble sugar analysis

Soluble sugar content of phloem, developing xylem, source and sink leaf tissue was analyzed following the procedure by Coleman *et al*. (2006) with some modifications. An aliquot (1 mL) of the upper phase from the extraction was collected and dried using a vacuum centrifuge. The pellet was resuspended in 1 mL of water and analyzed for sucrose, raffinose, galactinol and *myo*‐inositol on an Dionex HPLC fitted with a PA 200 (sucrose and raffinose) and a MA‐1 (*myo*‐inositol and galactinol) column (Dionex) with 600 mm NaOH at 0.5 mL/min. Fucose was added as internal standard.

### Starch analysis

The pellet retained from the soluble sugar isolation was dried overnight in a 50 °C oven and extracted according to Coleman *et al*. (2006), An aliquot of the supernatant was analyzed by HPLC to quantify glucose release, representing the starch that was hydrolyzed by the acid treatment, using the same conditions as described above.

### Cell wall characterization

Fibre length, wood density and microfibril angle were measured according to Ukrainetz *et al*. (2008) with some modifications. Fibre length was determined on a 1‐cm segment isolated from sections taken 10 cm above the root collar. Fibre length (in mm) was estimated for each sample by measuring 10 000 fibres. Wood density was determined in bark‐to‐bark samples isolated from sections taken 9 cm above the root collar. Density was measured on both sides of the pith and averaged for each sample.

Microfibril angle (MFA) and cell wall crystallinity were determined by X‐ray diffraction using a Bruker D8 Discover X‐ray diffraction unit. Both the X‐ray source and the detector were set to θ = 0° for MFA determination. The average T value of the two 002 diffraction arc peaks was used for MFA calculations, as per the method of Megraw *et al*. (1998). In contrast, the 2θ (source) was set to 17° for wood crystallinity determination. Crystallinity was determined by mathematically fitting the data using the method of Vonk (1973). Crystallinity measures were pre‐calibrated by capturing diffractograms of pure *A. xylinum* bacterial cellulose known to be 87% crystalline.

### Cross‐sectional staining and microscopy

Wood samples from 6‐month‐old *AtGolS3‐OE* trees were soaked overnight in dH_2_O. Samples were then cut into 40 μm cross‐sections with a Spencer AO860 hand sliding microtome (Spencer Lens Co., Buffalo, NY) and stored with dH_2_O until visualized. Sections were treated with 0.01% Calcofluor‐white in 1× PBS for 3 min, and then washed 3 × 5 min in 1× PBS for cellulose staining (Falconer and Seagull, 1985). Sections were also treated with a saturated solution of phloroglucinol in 10% HCl for lignin staining. Cross‐sections were treated with Lugol's solution (2% KI and 0.2% Iodine) to visualize starch. The sections were mounted onto glass slides and visualized with a Leica DRM microscope (Leica Microsystems, Wetzlar, Germany). Pictures were taken with a QICam camera (Q‐imaging, Surrey, BC) and analyzed with OpenLab 4.0Z software (PerkinElmer Inc., Waltham, MA).

Vessel number, length and area were calculated from the 40 μm cross‐sections stained with phloroglucinol. Three trees per line were analyzed. Pictures were taken in different zones of the sections and approximately 180 vessel areas were measured per tree. The sections were analyzed on the Carl Zeiss Jena ‘Jenamed’ 2 fluorescence microscope (Carl Zeiss Microscopy LLC, NY). Photos were taken with an Infinity 3 camera (Lumenera Corporation, Ottawa, ON) and analyzed with the Infinity capture program.

### Antibody labelling

The prepared cross‐section samples (described above) were subjected to antibody labelling. Briefly, nonspecific protein binding was blocked with 5% BSA in TBST (10 mm Tris‐buffer, 0.25 m NaCl, pH 7, with 0.1% Tween 20) for 20 min. Sections were then treated with diluted primary antibody (1:50), anti‐β‐(1–4)‐β‐mannan monoclonal antibody (catalogue #400‐4; Biosupplies Australia Pty Ltd, Melbourne, Australia), anti‐xylan LM10 antibody (kind gift of Dr. J. Paul Knox; www.plantprobes.co.uk) or CCRC‐M7 against RGI (Puhlmann *et al*., 1994) at room temperature for 1 h. The sections were then washed twice with TBST for 5 min. The diluted secondary antibody (Alexa 543: anti‐rat or anti‐mouse) 1:50 was added, incubated for 1 h and washed twice with TBST. Samples were mounted on glass slides with 90% glycerol or anti‐fade mounting media. Fluorescent localization was observed on a Leica DRM (Leica Microsystems) light microscope using a Texas Red filter, and images captured with a QICam camera (Qimaging) and analyzed with OpenLab 4.0Z software (Perkinelmer Inc.). The antibody‐tagged sections were stored at 4 °C in TBST 1× in microfuge tubes if they were not used immediately.

### NMR

NMR analysis was performed as described in the recent protocol (Mansfield *et al*., 2012). Briefly, plant biomass was air‐dried to a constant moisture content and cryogenically pre‐ground for 2 min at 30 Hz using a Retsch (Newtown, PA) MM301 mixer mill. Approximately, 30–60 mg of extract‐free, ball‐milled plant cell wall material was transferred to a 5‐mm NMR tube, and 500 μL of pre‐mixed DMSO‐d_6_/pyridine‐d_5_ (4:1) was added directly into the NMR tube containing individual samples. The NMR tubes were then placed in an ultrasonic bath and sonicated for 1–5 h, until the gel became homogeneous. 2D ^1^H–^13^C HSQC spectra were acquired using a standard Bruker pulse program (‘hsqcetgpsisp.2’ or ‘hsqcetgpsisp2.2’) Bruker Biospin (Billerica, MA) AVANCE 700 MHz spectrometer fitted with a cryogenically cooled 5‐mm QCI (^1^H/^31^P/^13^C/^15^N) gradient probe with inverse geometry (proton coils closest to the sample). The central residual DMSO peak was used as the internal reference (δ_C_, 39.52 ppm; δ_H_, 2.50 ppm). Interactive integrations of contours in 2D HSQC plots were carried out using Bruker's TopSpin 3.5 (Mac) software, as well as all data processing.

### Statistical analysis

Parametric or non‐parametric Student's *t*‐tests were performed (depending on equal or non‐equal variances). Student's *t*‐test values were calculated with Microsoft Excel (Microsoft Corp., WA). Significant differences were calculated between wild‐type trees and transgenic lines at the 95% confidence level.

### RNA purification, library construction and sequencing

RNA‐Seq data were analyzed from cambium/bark, developing xylem and source leaf tissue of 5‐month‐old wild type and *AtGolS3‐OE* poplar (line 6). Tissue was ground on mortar and pestle under liquid N. RNA was extracted and purified using the PureLink^®^ Plant RNA Reagent (Invitrogen) following the manufacturer's protocol. Subsequently, RNA was purified using RNeasy Plant Mini Kit (Qiagen Inc., Toronto, ON) with the RNA Cleanup and On‐Column DNase digestion steps following the manufacturer's protocol. RNA quantification and quality were measured with a 2100 BioAnalyzer instrument (Agilent Technologies, Santa Clara, CA). Samples with RIN (RNA integrity number) above 7 were processed at the Michael Smith Genome Sciences Centre (Vancouver, BC) where the library was assembled and the transcriptome sequenced. Sequencing was carried out on an Illumina HiSeq instrument (Illumina Inc., San Diego, CA).

### mRNA‐Seq data processing and quality control

The raw RNA‐Seq data were analyzed using a local Galaxy pipeline (http://usegalaxy.org) as described by Hefer *et al*. (2015) with some modifications. Paired end reads were trimmed to remove low‐quality reads and adapters using Trimmomatic (reads with average quality of 20 were kept and reads shorter than 50 bp were discarded; Bolger *et al*., 2014). Tophat v. 2.0.8 (Trapnell *et al*., 2012) was then used to map the trimmed reads to v3.0 of the *P. trichocarpa* genome (www.phytozome.com). Cufflinks v2.1.1 (Trapnell *et al*., 2012) was used to calculate fragments per kilobase of transcript per million mapped reads (FPKM) values for each transcript. MapMan (http://mapman.gabipd.org/web/guest/home) was used to visualize the data.

## Supporting information


**Figure S1.** MapMan visualization of the transcriptional response of poplar to the overexpression of the *AtGolS3*.

**Table S1.** Differentially expressed genes in transgenic poplar overexpressing AtGolS3 poplar leaves.
