# Supplementary material for: Altering carbon allocation in hybrid poplar (Populus alba × grandidentata) impacts cell wall growth and development
Source: Plant Biotechnol J. 2017 Mar 4;15(7):865–78. doi: 10.1111/pbi.12682 (PMC5466441; doi:10.1111/pbi.12682)
Supplement: Supplementary file 1 — Figure S1. MapMan visualization of the transcriptional response of poplar to the overexpression of the AtGolS3. [file PBI-15-865-s002.docx]

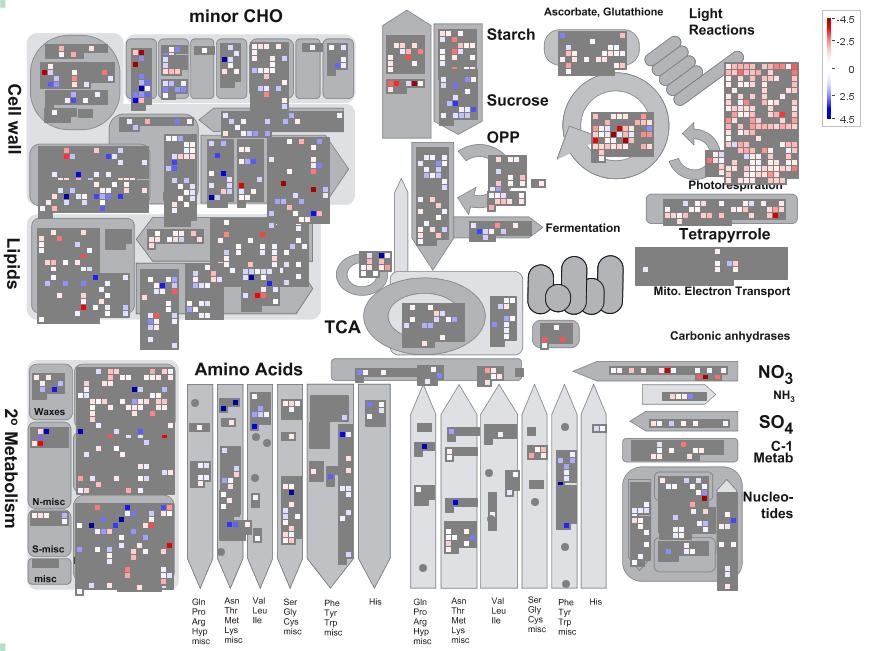


**Supplemental Figure 1.** MapMan visualization of the transcriptional response of poplar to the overexpression of the *AtGolS3*. Schematic overview of the changes in metabolism-related gene expression in source leaves of *AtGolS3* overexpressing poplar (line 6) compared to wild-type tree. Heat map representation indicates log2 -fold changes. Blue: upregulated. Red: downregulated. Red arrow indicates photosynthesis and photorespiration- related genes. Blue arrows indicate sucrose and *myo*-inositol metabolism-related genes.
